# High-capacity dilithium hydroquinone cathode material for lithium-ion batteries

**DOI:** 10.1093/nsr/nwae146

**Published:** 2024-04-16

**Authors:** Yong Lu, Haoqin Han, Zhuo Yang, Youxuan Ni, Zhicheng Meng, Qiu Zhang, Hao Wu, Weiwei Xie, Zhenhua Yan, Jun Chen

**Affiliations:** Frontiers Science Center for New Organic Matter, Key Laboratory of Advanced Energy Materials Chemistry (Ministry of Education), State Key Laboratory of Advanced Chemical Power Sources, College of Chemistry, Nankai University, Tianjin 300071, China; Frontiers Science Center for New Organic Matter, Key Laboratory of Advanced Energy Materials Chemistry (Ministry of Education), State Key Laboratory of Advanced Chemical Power Sources, College of Chemistry, Nankai University, Tianjin 300071, China; Frontiers Science Center for New Organic Matter, Key Laboratory of Advanced Energy Materials Chemistry (Ministry of Education), State Key Laboratory of Advanced Chemical Power Sources, College of Chemistry, Nankai University, Tianjin 300071, China; Frontiers Science Center for New Organic Matter, Key Laboratory of Advanced Energy Materials Chemistry (Ministry of Education), State Key Laboratory of Advanced Chemical Power Sources, College of Chemistry, Nankai University, Tianjin 300071, China; Frontiers Science Center for New Organic Matter, Key Laboratory of Advanced Energy Materials Chemistry (Ministry of Education), State Key Laboratory of Advanced Chemical Power Sources, College of Chemistry, Nankai University, Tianjin 300071, China; Frontiers Science Center for New Organic Matter, Key Laboratory of Advanced Energy Materials Chemistry (Ministry of Education), State Key Laboratory of Advanced Chemical Power Sources, College of Chemistry, Nankai University, Tianjin 300071, China; Frontiers Science Center for New Organic Matter, Key Laboratory of Advanced Energy Materials Chemistry (Ministry of Education), State Key Laboratory of Advanced Chemical Power Sources, College of Chemistry, Nankai University, Tianjin 300071, China; Frontiers Science Center for New Organic Matter, Key Laboratory of Advanced Energy Materials Chemistry (Ministry of Education), State Key Laboratory of Advanced Chemical Power Sources, College of Chemistry, Nankai University, Tianjin 300071, China; Frontiers Science Center for New Organic Matter, Key Laboratory of Advanced Energy Materials Chemistry (Ministry of Education), State Key Laboratory of Advanced Chemical Power Sources, College of Chemistry, Nankai University, Tianjin 300071, China; Frontiers Science Center for New Organic Matter, Key Laboratory of Advanced Energy Materials Chemistry (Ministry of Education), State Key Laboratory of Advanced Chemical Power Sources, College of Chemistry, Nankai University, Tianjin 300071, China

**Keywords:** lithium-ion batteries, cathode materials, lithiated organic materials, dilithium hydroquinone, separator modification

## Abstract

Lithiated organic cathode materials show great promise for practical applications in lithium-ion batteries owing to their Li-reservoir characteristics. However, the reported lithiated organic cathode materials still suffer from strict synthesis conditions and low capacity. Here we report a thermal intermolecular rearrangement method without organic solvents to prepare dilithium hydroquinone (Li_2_Q), which delivers a high capacity of 323 mAh g^−1^ with an average discharge voltage of 2.8 V. The reversible conversion between orthorhombic Li_2_Q and monoclinic benzoquinone during charge/discharge processes is revealed by *in situ* X-ray diffraction. Theoretical calculations show that the unique Li–O channels in Li_2_Q are beneficial for Li^+^ ion diffusion. *In situ* ultraviolet-visible spectra demonstrate that the dissolution issue of Li_2_Q electrodes during charge/discharge processes can be handled by separator modification, resulting in enhanced cycling stability. This work sheds light on the synthesis and battery application of high-capacity lithiated organic cathode materials.

## Teaser

The lithiated organic cathode material, dilithium hydroquinone, has been synthesized by a facile thermal intermolecular rearrangement method, and shows a good promise as high-capacity cathodes for lithium-ion batteries.

## INTRODUCTION

Lithium-ion batteries (LIBs) have dominated the market of portable electronics and shown great promise for large-scale energy storage applications since their commercialization in the early 1990s [1,2]. The current electrochemistry of cathode materials in commercial LIBs is based on Li-ion interaction/de-interaction in transition-metal oxides or phosphates [3–5]. The limitations of inorganic cathode materials mainly include scarce natural resources, recyclability issues, and high CO_2_ emissions and energy consumption during the material production process [6–8]. Thus, the development of new cathode materials is urgently needed. In recent years, organic electrode materials have attracted much attention because of their high abundance, environmental friendliness and renewability [9–11]. Unfortunately, the reported organic cathode materials are mostly free of Li initially and used at oxidation states, which have to match with Li-containing anodes such as Li metal [12–15]. The immaturity of Li-containing anodes inevitably hinders the practical application of most organic cathode materials. In this regard, developing lithiated organic cathode materials at reduced states as Li reservoirs to match with commercial Li-free anode materials, such as graphite, is of great significance for future commercial applications.

In fact, the investigation of lithiated organic cathode materials is still in its infancy [16]. The most widely studied lithiated organic cathode materials are dilithium (2,5-dilithium-oxy)-terephthalate (Li_4_-*p*-DHT) and its derivatives [17–22]. Li_4_-*p*-DHT can be prepared by the reaction between CH_3_OLi and 2,5-dihydroxyterephthalic acid in absolute methanol, showing a discharge capacity of 223 mAh g^−1^ at 0.1 C after morphology optimization [17]. The capacity is lower for the derivatives of Li_4_-*p*-DHT owing to the introduction of inactive atoms/groups [19,20,22]. Recently, Vlad's group prepared a series of lithiated organic cathode materials based on conjugated sulfonamides by using CH_3_OLi and LiH as lithiation reagents [23,24]. Among them, dilithium 1,4-phenylenebis((methylsulfonyl)amide) exhibits the highest reversible capacity, ∼166 mAh g^−1^. Similarly, the same group subsequently developed families of conjugated triflimides and cyanamides as high-voltage lithiated cathode materials by using CH_3_OLi and LiH as lithiation reagents [25]. In addition, other types of lithiated organic cathode materials based on different active sites such as C=N–Li and N–O–Li were also reported [26,27]. For example, lithium tetracyanoquinodimethane featuring active groups of C=N–Li was demonstrated to be electrochemically reversible, however, with a low capacity of 127 mAh g^−1^ [26]. To date, the most common lithiation reagents used to synthesize lithiated organic cathode materials have been LiH and CH_3_OLi, which are very chemically reactive and hard to apply to large-scale practical applications. Moreover, the reversible capacities of lithiated organic cathode materials are generally limited. In short, the strict synthesis conditions derived from the use of LiH or CH_3_OLi and the limited reversible capacity are the two common challenges facing lithiated organic cathode materials.

Herein, we develop a thermal intermolecular rearrangement method under mild conditions to realize the scalable synthesis of the lithiated organic cathode material dilithium hydroquinone (Li_2_Q), which shows a high reversible capacity of 323 mAh g^−1^ with an average discharge voltage of 2.8 V at 0.1 C, corresponding to an energy density of ∼900 Wh kg^−1^ at the active material level. The pristine Li_2_Q with an orthorhombic structure converts to 1,4-benzoquinone (BQ) with a monoclinic structure after charge, and the conversion is reversible during the subsequent discharge process, as revealed by *in situ* X-ray diffraction (XRD) patterns. Density functional theory (DFT) calculations found that there are unique Li–O channels for facile Li^+^ ion diffusion in Li_2_Q. The cycling stability of Li_2_Q can be improved by separator optimization owing to the mitigated dissolution issue, as observed via *in situ* ultraviolet-visible (UV-vis) spectroscopy. This work illustrates the facile synthesis and battery applications of lithiated organic cathode materials.

## RESULTS AND DISCUSSION

**Figure 1.**
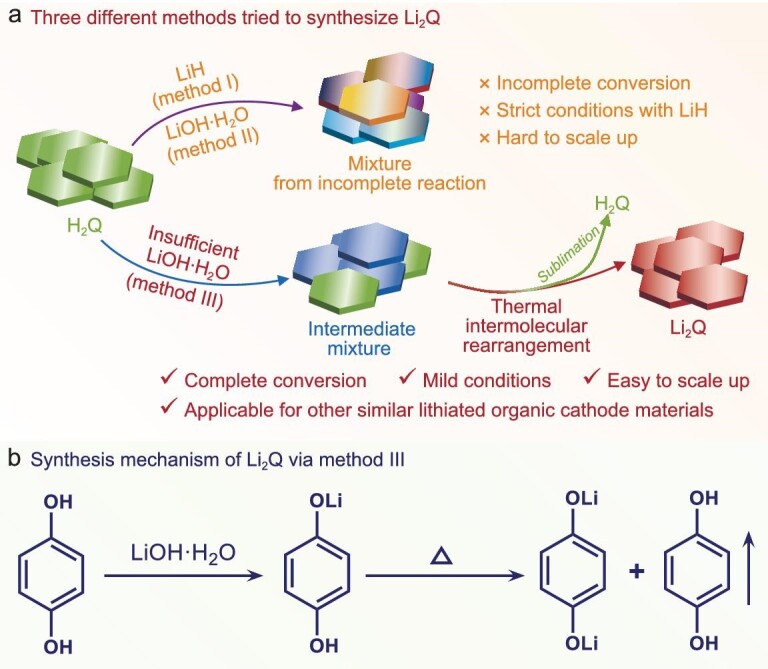
Synthesis of the lithiated organic cathode material Li_2_Q. (a) Schematic diagrams of three different methods used to synthesize Li_2_Q and the corresponding characteristics. (b) Synthesis mechanism of obtaining pure Li_2_Q by using insufficient LiOH·H_2_O to react with H_2_Q, and then annealing to generate Li_2_Q.

Three different approaches have been tried to prepare Li_2_Q from hydroquinone (H_2_Q), as shown in Fig. 1a. The first method utilizes LiH as the lithiation reagent and anhydrous 1,2-dimethoxyethane (DME) as the solvent under conditions free of H_2_O and O_2_. After adequate stirring, the DME solvent was evaporated in a vacuum to obtain the product. The infrared (IR) spectra and ^1^H nuclear magnetic resonance (NMR) spectra of the pristine H_2_Q and obtained product are shown in Figs S1 and S2 (Supplementary Data), respectively. The results show that there are obvious peaks assigned to the stretching vibration of residual O–H, indicating that the neutralization reaction between H_2_Q and LiH cannot proceed completely. Moreover, the industrial production of LiH is challenging and dangerous because it generally comes from the reaction between Li metal and H_2_ at high temperature and/or high pressure [28]. We then used LiOH·H_2_O as the lithiation reagent under Ar atmosphere with H_2_O as the solvent. The IR and ^1^H NMR spectra of the obtained product in Figs S3 and S4 indicate that there is also residual O–H and the neutralization reaction between H_2_Q and LiOH is incomplete. This could be attributed to the weak dissociation of the second H^+^ of H_2_Q (pK_a2_ = 11.4) [29]. To sum up, it is hard to synthesize pure Li_2_Q using the two aforementioned methods (Fig. 1a). Thus, new approaches for synthesizing pure Li_2_Q are urgently needed.

The incomplete reactions between H_2_Q and LiOH·H_2_O lead to the emergence of monolithium hydroquinone (LiHQ). On the basis of the intermolecular interaction of LiHQ and the sublimation property of H_2_Q, we develop a new method, thermal intermolecular rearrangement, to synthesize pure Li_2_Q. As shown in Fig. 1a, we firstly used excess H_2_Q to react with insufficient LiOH·H_2_O in water under Ar atmosphere, to generate the mixture containing LiHQ. Then, the mixture was annealed at 180°C to make LiHQ decompose to Li_2_Q and H_2_Q. All H_2_Q escapes from this system in a gas state (Fig. 1b). The sublimated material is indeed pure H_2_Q, as confirmed by IR and ^1^H NMR spectra (Figs S5 and S6). The thermal intermolecular rearrangement reaction is entropy-increasing and thus easy to carry out, and is similar to the thermal decomposition of NaHCO_3_ to Na_2_CO_3_ and H_2_CO_3_ (i.e. CO_2_, H_2_O). The synthesis of Li_2_Q by this method is green because no organic solvent was used during the reaction and isolation processes. This is superior to the previously reported methods of synthesizing lithiated organic cathode materials using LiH or CH_3_OLi with regard to toxicity, cost and safety. More importantly, this method is generalizable and could be extended to synthesize other similar lithiated organic cathode materials. The basic prerequisite of this method, used for other lithiated organic materials, is that the raw material (phenol) can sublimate at a proper temperature before the decomposition of the target product.

**Figure 2.**
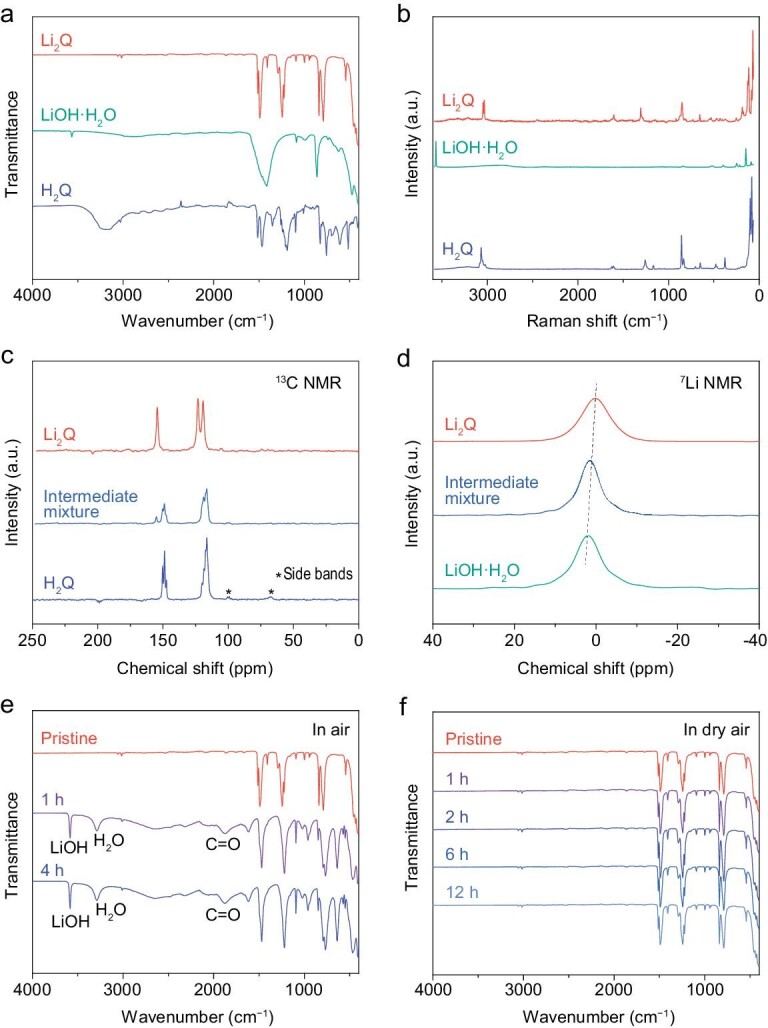
Characterization of Li_2_Q. (a) IR and (b) Raman spectra of H_2_Q, LiOH·H_2_O and Li_2_Q. (c) Solid-state ^13^C NMR spectra of H_2_Q, the intermediate mixture and Li_2_Q. (d) Solid-state ^7^Li NMR spectra of LiOH·H_2_O, the intermediate mixture and Li_2_Q. The evolution of IR spectra of Li_2_Q at different exposure times (e) in ambient air and (f) in dry air (dew point: about −40°C).

The product obtained by the thermal intermolecular rearrangement method was characterized by various approaches (Fig. 2). The IR spectra of H_2_Q, LiOH·H_2_O and the product in Fig. 2a indicate that there is no peak assigned to O–H of H_2_Q or LiOH·H_2_O, implying the complete substitution of H in O–H of H_2_Q by Li. The results can also be confirmed by the Raman spectra of H_2_Q, LiOH·H_2_O and the product (Fig. 2b). The liquid-state ^1^H NMR spectrum of the Li_2_Q product is shown in Fig. S7. Except for the solvent peak, no other peaks can be observed, implying that the solubility of Li_2_Q in deuterium dimethyl sulfoxide (DMSO-*d6*) with high polarity is limited. Thus, we used solid-state NMR to further characterize the raw material, intermediate mixture and products. The solid-state ^13^C NMR spectra in Fig. 2c show that there are two types of C in H_2_Q, that is, 149 ppm (C of C–OH) and 116 ppm (other C of the benzene ring). From the intermediate mixture to Li_2_Q, the peak belonging to C of C–OH disappears completely and meanwhile the peak at 154 ppm, which can be assigned to C of C–OLi, emerges, suggesting the successful synthesis of Li_2_Q. Furthermore, we obtained the solid-state ^7^Li NMR spectra of LiOH·H_2_O, intermediate mixture and Li_2_Q. The results in Fig. 2d show that Li_2_Q has the lowest chemical shift of −0.947 ppm when compared with LiOH·H_2_O and the intermediate mixture.

For practical applications, the air stability, thermal stability and electronic conductivity of cathode materials are very important, especially for lithiated organic materials at reduced states. Figure 2e shows the evolution of the IR spectra of Li_2_Q in ambient air. The results indicate that Li_2_Q tends to absorb H_2_O in air, which leads to hydrolysis and oxidation, as proved by the appearance of peaks attributed to H_2_O, LiOH and C=O after exposure for 1 h and 4 h. This is the reason why the synthetic process was conducted in Ar atmosphere instead of air. In contrast, the stability of Li_2_Q in dry air (dew point: about −40°C) is much better, as confirmed by the IR spectra in Fig. 2f, where no change was observed after being placed for 12 h. The high stability of Li_2_Q in dry air means it can be handled and processed in practical applications. We also evaluated the thermal stability of Li_2_Q, which affects the safety of batteries. The weight loss of Li_2_Q in Ar atmosphere starts at ∼568°C, implying its high thermal stability (Fig. S8). Moreover, the electronic conductivity of Li_2_Q tested via digital multimeter is 9.37 × 10^−10^ S cm^−1^, which is close to that of inorganic LiFePO_4_ cathode material [30]. In addition, the scanning electron microscopy (SEM) images of Li_2_Q show that it exists in the form of micrometer-level particles (Fig. S9).

**Figure 3.**
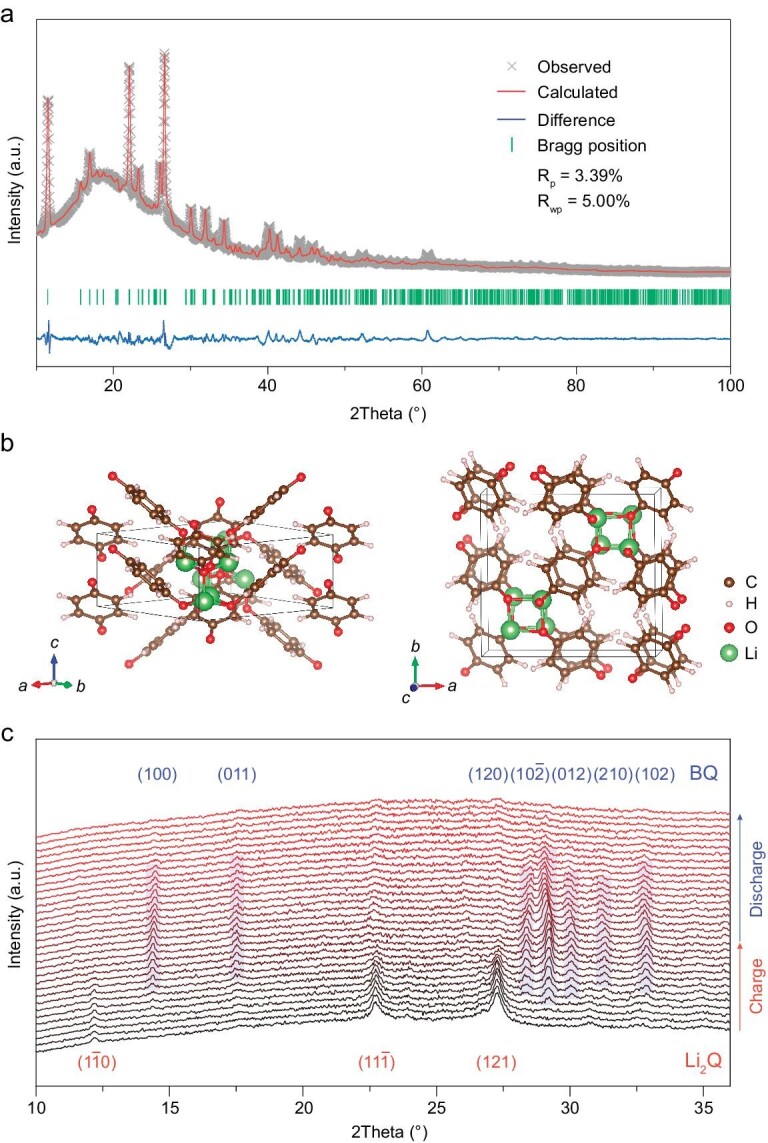
Crystal structure of Li_2_Q and evolution during charge and discharge processes. (a) XRD pattern and corresponding Rietveld refinement, and (b) crystal structure with different views of Li_2_Q. (c) *In situ* XRD patterns of Li_2_Q in the first cycle, where the main diffraction peaks and corresponding crystal planes of Li_2_Q and charge product BQ are marked.

We then studied the crystal structure of the obtained Li_2_Q through XRD testing and theoretical calculations. At first, we optimized the crystal structure of Li_2_Q through theoretical calculations. Then, Rietveld refinement was conducted to simulate the experimental XRD pattern, as shown in Fig. 3a. The results reveal that the crystal system of Li_2_Q is orthorhombic. Detailed crystal parameters can be seen in Table S1 (Supplementary Data). The three main peaks at 2*θ* = 11.7°, 22.2° and 26.7° are assigned to the crystal plane of (1$\bar{1}$0), (11$\bar{1}$) and (121) of Li_2_Q, respectively. Figure 3b shows the crystal structure of Li_2_Q with different views, where Li atoms are fixed by the neighboring three organic molecules and the length of the Li–O bond is ∼2.0 Å. To the best of our knowledge, this is the first time the crystal structure of Li_2_Q has been obtained.

The crystal structure evolution of Li_2_Q during charge and discharge processes was further investigated by *in situ* XRD. As shown in Fig. 3c, the three main diffraction peaks attributed to the crystal plane of (1$\bar{1}$0), (11$\bar{1}$) and (121) of Li_2_Q can be detected easily in the pristine electrode. These peaks gradually decrease and disappear completely after charging to 3.5 V (vs. Li^+^/Li). Meanwhile, the diffraction peaks at 2*θ* = 14.4°, 17.5°, 28.4°, 29.2°, 30.0°, 31.3° and 32.9° gradually emerge and become stronger and stronger with the charge process. As shown in Figs S10 and S11, these peaks are well consistent with the crystal plane of (100), (011), (120), (10$\bar{2}$), (012), (210) and (102) of BQ, featuring a monoclinic crystal system, respectively. During the subsequent discharge process, these peaks of BQ gradually disappear. Meanwhile, the two main peaks at 2*θ* = 22.2° and 26.7° assigned to the crystal plane of (11$\bar{1}$) and (121) of Li_2_Q emerge but are weak after being fully discharged, implying that the crystallinity of Li_2_Q after the first cycle decreases when compared with pristine Li_2_Q. In subsequent cycles, the crystallinity of the charge product (BQ) does not change significantly and only becomes slightly weak when compared with the first cycle (Fig. S12), which could be attributed to the dissolution of BQ in electrolyte. In contrast, the crystallinity of the discharge product (Li_2_Q) in subsequent cycles remains weak and almost unchanged when compared with the first cycle. These results demonstrate the reversible conversion between orthorhombic Li_2_Q with low crystallinity and monoclinic BQ with relatively high crystallinity during repeated charge and discharge processes after the first cycle. The lower crystallinity of Li_2_Q than BQ during cycles may be ascribed to the fact that the latter is arranged in the same orientation (Fig. S11).

**Figure 4.**
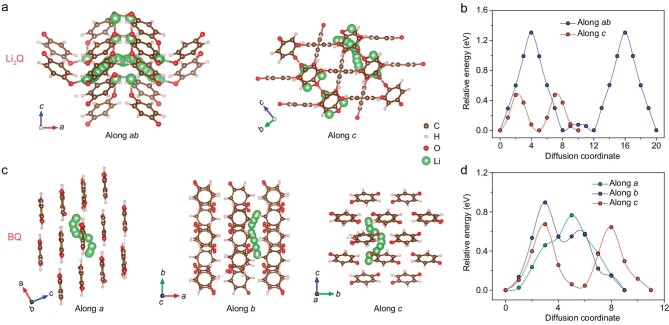
Li^+^ ion diffusion behaviors in Li_2_Q and BQ. (a) The calculated different Li^+^ ion diffusion pathways and (b) corresponding relative energy of each pathway of Li_2_Q. (c) The calculated different Li^+^ ion diffusion pathways and (d) corresponding relative energy of each pathway of BQ.

After revealing the crystal structure evolution of Li_2_Q during cycles, we used DFT calculations to study the accessible Li^+^ ion diffusion pathways in the crystal structure of pristine Li_2_Q and the charge product BQ. As shown in Fig. 4a, two diffusion pathways, i.e. along *ab* and along *c* directions, are included in Li_2_Q based on its bulk structure. The diffusion of Li^+^ ion along the *ab* direction goes through the interlayer of benzene rings. Due to the weak interaction between benzene rings and Li^+^ ions, the diffusion energy barrier along the *ab* direction is as high as 1.31 eV (Fig. 4b). The diffusion of Li^+^ ion along the *c* direction is along the channel composed of O atoms, and this unique Li–O channel enables facile Li^+^ diffusion through the Li_2_Q crystal structure (Fig. 4a). Thus, the diffusion along the *c* direction shows a lower energy barrier of 0.47 eV, suggesting that the Li^+^ ion diffusion along the *c* direction is more favorable than that along the *ab* direction (Fig. 4b).

For the charge product BQ, there are three possible Li^+^ ion diffusion pathways, that is, along *a, b* and *c* directions (Fig. 4c). The diffusion of Li^+^ ion along the *a* direction goes through the organic layers composed of benzene rings. The diffusions of Li^+^ ion along the *b* and *c* directions are both along the interlayer between organic layers. As shown in Fig. 4d, the diffusion energy barriers along *a, b* and *c* directions are 0.77 eV, 0.90 eV and 0.67 eV, respectively. Among them, the Li^+^ diffusion along the *c* direction in BQ is the most favorable one, and similar to the result in Li_2_Q. When compared with other organic electrode materials in previous works [31,32], the Li^+^ ion diffusion energy barriers in Li_2_Q and BQ along the *c* direction are relatively low, implying the effective diffusion of Li^+^ ions through both Li_2_Q and BQ. This might explain the high rate performance of batteries, which will be discussed later.

**Figure 5.**
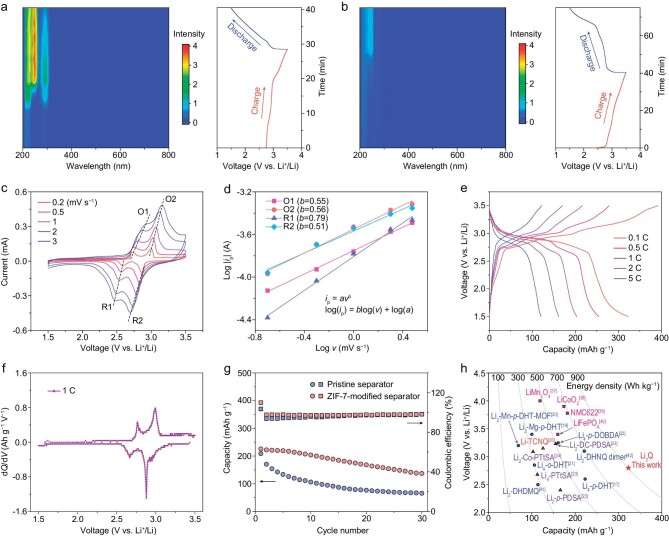
Electrochemical behaviors of Li_2_Q. *In situ* UV-vis spectra (color-mapped profiles) of Li_2_Q batteries during charge and discharge processes by using (a) a pristine separator and (b) a ZIF-7-modified separator. (c) CV curves at different scan rates (0.2, 0.5, 1, 2 and 3 mV s^−1^) and (d) the corresponding observed and fitting plots of log(|*i*_p_|) versus log(*ν*) at the four peak currents. (e) Typical charge/discharge curves at different current rates (0.1, 0.5, 1, 2 and 5 C) and (f) the corresponding differential capacitance *dQ*/*dV* plot of the charge/discharge curves at 1 C. (g) Cycling performance (discharge capacity and Coulombic efficiency) at 1 C with pristine and ZIF-7-modified separators. (h) Comparison of capacity, voltage and corresponding energy density of Li_2_Q, commercial inorganic cathode materials and other representative lithiated organic cathode materials prepared by chemical methods.

One of the major issues facing most organic electrode materials is their high solubility, and/or the high solubility of redox intermediates in electrolyte, leading to a shuttle effect and poor cycling stability [33,34]. Thus, prior to the electrochemical performance test, we used *in situ* UV-vis spectra to investigate the dissolution behavior of Li_2_Q electrodes in electrolyte during charge and discharge processes. The *in situ* UV-vis spectra tests were conducted using a homemade cell with ∼3 mL electrolyte (Fig. S13). As shown in Fig. 5a and Fig. S14a, pristine Li_2_Q shows limited dissolution in 1 mol kg^−1^ lithium bis(trifluoromethanesulfonyl) imide (LiTFSI) dissolved in ethylene carbonate and dimethyl carbonate (EC:DMC = 1:1 vol%) electrolyte. However, the solubility gradually increases with the charging process, especially at the end of charging (3.5 V vs. Li^+^/Li). In the following discharge process, the UV-vis spectra do not change obviously, implying that the dissolved charge products cannot be fully utilized during the discharge process.

To address the dissolution problem, we used a metal-organic framework (zeolitic imidazolate framework-7, ZIF-7) as the modification layer on the Celgard separator because ZIF-7 shows a small pore size of only ∼2.9 Å, which can block active materials [35]. The ZIF-7 material was prepared in aqueous solution and its XRD pattern in Fig. S15 is in agreement with the result of previous reports [35,36]. Moreover, the obtained ZIF-7 exists in the form of micrometer-level cuboid particles, as seen in the SEM images (Fig. S16). The SEM images of the ZIF-7-modified separator in Fig. S17 show that ZIF-7 particles are distributed uniformly on the separator. In addition, the cross-sectional SEM image indicates that the thickness of the ZIF-7-based layer on the separator is ∼10 μm (Fig. S18). The *in situ* UV-vis spectra of a Li_2_Q battery using the ZIF-7-modified separator to fully wrap the Li_2_Q electrode during charge and discharge processes are shown in Fig. 5b and Fig. S14b. The results suggest that the dissolution of the electrode in electrolyte during charge and discharge processes can be suppressed effectively when compared with a pristine separator. Thus, we select the ZIF-7-modified Celgard as the separator for electrochemical performance studies.

As shown in Fig. 5c, cyclic voltammetry (CV) at different scan rates (0.2, 0.5, 1, 2 and 3 mV s^−1^) of Li_2_Q indicates that there are two distinct redox couples (O1/R1, O2/R2). The fitting results of the four redox peak currents, log(|*i*_p_|), versus scan rate, log(*ν*), are shown in Fig. 5d. The four *b* values are confirmed to be 0.56, 0.51, 0.55 and 0.79 for the peaks of O2, R2, O1 and R1, respectively. The results suggest that the electrochemical redox process of Li_2_Q is primarily controlled by Li^+^ ion diffusion.

Subsequently, we further investigated the electrochemical performance of Li_2_Q. Figure 5e shows the charge/discharge curves at different current rates (0.1, 0.5, 1, 2 and 5 C). The corresponding differential capacitance dQ/dV plot of the charge/discharge curves at 1 C is provided in Fig. 5f, which is consistent with the CV results in Fig. 5c. The discharge capacities of Li_2_Q are 323, 255, 204, 162 and 120 mAh g^−1^ at 0.1, 0.5, 1, 2 and 5 C, respectively (Fig. 5e). Moreover, the battery with a ZIF-7-modified separator exhibits improved cycling performance with a capacity retention of 61% after 30 cycles at 1 C, which is much higher than with a pristine separator under the same test conditions (32%), demonstrating the positive effect of a ZIF-7-modified separator with regard to the stability of Li_2_Q during cycles (Fig. 5g). Compared with other reported lithiated organic cathode materials, Li_2_Q in this work exhibits an outstanding capacity of 323 mAh g^−1^ and a high energy density of ∼900 Wh kg^−1^ at 0.1 C. The capacity and energy density of Li_2_Q are higher than those of not only commercial inorganic cathode materials [37–40], but also most reported lithiated organic cathode materials prepared by chemical methods (Fig. 5h) [17,19–26,41,42]. Together with the developed facile synthesis method under mild conditions that has been demonstrated in this work, Li_2_Q shows great promise for battery applications. However, although progress has been made in this work, the cycling stability of Li_2_Q still needs to be further improved through more elaborate efforts in the future, with the aim of achieving the practical application of Li_2_Q.

## CONCLUSIONS

In summary, we have successfully developed a thermal intermolecular rearrangement method to prepare the lithiated organic cathode material Li_2_Q. A combination of theoretical calculations and *in situ* XRD studies reveals that pristine Li_2_Q with an orthorhombic structure converts to BQ with a monoclinic structure after charge, and the conversion is reversible during the subsequent discharge process. Further theoretical calculations found that the diffusion of Li^+^ ion along the *c* direction is along the channel composed of O atoms, which enables facile Li^+^ ion diffusion through the Li_2_Q crystal structure. As a cathode material, Li_2_Q can deliver a high capacity of 323 mAh g^−1^ with an average discharge voltage of 2.8 V. *In situ* UV-vis spectra indicate that the dissolution issue of Li_2_Q electrodes during charge and discharge processes can be effectively mitigated by the ZIF-7-modified separator, resulting in enhanced cycling stability. This work paves the way for promoting the facile synthesis and battery applications of high-capacity lithiated organic cathode materials.

## Supplementary Material

nwae146_Supplemental_File
