# Supplementary material for: High-capacity dilithium hydroquinone cathode material for lithium-ion batteries
Source: Natl Sci Rev. 2024 Apr 16;11(6):nwae146. doi: 10.1093/nsr/nwae146 (PMC11089817; doi:10.1093/nsr/nwae146)
Supplement: nwae146_Supplemental_File [file nwae146_supplemental_file.pdf]

Supplementary Information

High-capacity dilithium hydroquinone cathode material for lithium-ion batteries

Yong Lu[+]*, Haoqin Han*[+]*, Zhuo Yang*[+]*, Youxuan Ni, Zhicheng Meng, Qiu Zhang, Hao Wu, Weiwei Xie, Zhenhua Yan, and Jun Chen**

Frontiers Science Center for New Organic Matter, Key Laboratory of Advanced Energy Materials Chemistry (Ministry of Education), State Key Laboratory of Advanced Chemical Power Sources, College of Chemistry, Nankai University, Tianjin 300071, China

[+]These authors contributed equally to this work

[*]Correspondence: chenabc@nankai.edu.cn

Table of Contents

Experimental Section······S3

Density Functional Theory (DFT) Calculation Details······S6

Figure S1. IR spectra of H_2Q and the product obtained via the first method······S7

Figure S2. ^{1}H NMR spectra of H_2Q and the product obtained via the first method······S7

Figure S3. IR spectrum of the product obtained via the second method······S8

Figure S4. ^{1}H NMR spectrum of the product obtained via the second method······S8

Figure S5. IR spectrum of the sublimated material······S9

Figure S6. ^{1}H NMR spectrum of the sublimated material······S9

Figure S7. Liquid-state ^{1}H NMR spectrum of Li_2Q······S10

Figure S8. TG curve of the prepared Li_2Q······S10

Figure S9. SEM images with low and high resolution of the prepared Li_2Q······S11

Table S1. Crystal parameters of Li_2Q······S11

Figure S10. XRD pattern of the charge product in the initial cycle······S12

Figure S11. Crystal structures of the charge product according to Rietveld refinement······S12

Figure S12. XRD patterns of the fully charged and discharged products in the 4th cycle······S13

Figure S13. Optical photograph of the homemade cell for in situ UV-vis spectra tests······S13

Figure S14. Detailed curves of the in situ UV-vis spectra······S14

Figure S15. XRD pattern of the prepared ZIF-7······S14

Figure S16. SEM images with low and high resolution of the prepared ZIF-7······S15

Figure S17. SEM images of the ZIF-7 modified Celgard separator······S15

Figure S18. Cross-sectional SEM image of the ZIF-7 modified Celgard separator······S16

References······S17

Experimental Section

Three different methods tried to synthesize Li_2Q

The first method (method I in Fig. 1a) with LiH as the lithiation reagent. This method was conducted in Ar-filled glove box. Hydroquinone (H_2Q, Sigma-Aldrich, 330 mg, 3 mmol) was dissolved in 10 mL anhydrous 1,2-dimethoxyethane (DME, DoDoChem). After stirring for complete dissolution, LiH (Innochem, 48 mg, 6 mmol) was added under slowly stirring. Then, the suspension was stirred continuously for 4 hours at room temperature. After that, DME in the suspension was evaporated by using vacuum pump to generate solid sample, which was then heated at 100 °C for 12 hours under vacuum to remove the residual DME to obtain the final product.

The second method (method II in Fig. 1a) with excess $LiOH·H_2O$ as the lithiation reagent. This method was conducted in Ar-filled H_2O-containing glove box. $LiOH·H_2O$ (Aladdin, 6.3 g, 0.15 mol) was dissolved in 50 mL anaerobic water which was prepared by freezing a water-filled flask in liquid nitrogen bath and unfreezing under vacuum for repeated three times. Upon $LiOH·H_2O$ completely dissolved, H_2Q (5.5 g, 0.05 mol) was added under continuous stirring. After stirring for 6 hours at room temperature, the suspension was filtered and the filter cake was washed by anaerobic water. Then, the sample was heated at 150 °C for 12 hours under vacuum to remove the residual water to obtain the final product.

The third method (method III in Fig. 1a) with insufficient $LiOH·H_2O$ as the lithiation reagent and excess H_2Q (thermal intermolecular rearrangement method). $LiOH·H_2O$ (4.2 g, 0.1 mol) was dissolved in 50 mL anaerobic water. Upon $LiOH·H_2O$ completely dissolved, H_2Q (16.5 g, 0.15 mol) was added under continuous stirring. After stirring for 4 hours under Ar atmosphere at room temperature, H_2O in the suspension was evaporated by using vacuum pump and meanwhile heating at 50 °C to generate solid sample (the intermediate mixture). The final product can be obtained by heating the intermediate mixture at 180 °C in Ar-filled glove box

until there is no sublimation. The sublimation (proven to be H_2Q) was collected, and can be reused. The Li_2Q product yield by this method is ~95.6% based on the amount of $LiOH{\cdot}H_2O$.

Synthesis of ZIF-7

ZIF-7 was prepared according to the previous work.[1] At first, 1.58 g $Zn(CH_3COO)_2{\cdot}2H_2O$ was dissolved in 900 mL deionized water. Then, 3.54 g benzimidazole was added. After stirring for 3 hours at room temperature, the suspension was aging for 24 hours. The solid sample was collected by centrifuging the suspension at 10000 rpm for 5 minutes. Then, the solid sample was washed by deionized water and methanol under centrifugation for three times, followed by soaked in methanol for 3 days and then heated at 120 °C for 12 hours in vacuum oven to generate the final ZIF-7 powder product.

Preparation of ZIF-7 modified separator

ZIF-7 powder and conductive carbon (Super P) with a mass ratio of 8:1 were mixed uniformly with a mortar. The polyvinylidene fluoride (PVDF) binder in NMP solution (10 wt%) was added with the mass of PVDF equal to that of Super P, namely, the mass ratio of ZIF-7, Super P, and PVDF is 8:1:1. Then, a few drops of NMP were added and the mixture was homogenized for 20 minutes. The resulting slurry was cast on Celgard separator. The separator was then heated at 60 °C for 24 hours under vacuum and cut into a circular disc with a diameter of 18 mm for battery fabrication.

Materials characterizations

Infrared spectrum (IR) was recorded by Brucker Tensor II, ATR mode. Liquid nuclear magnetic resonance (NMR) was collected by Brucker AVANCE III 400 MHz. The trace amount of H_2O in DMSO-*d6* was fully removed by activated molecular sieve before used for NMR tests. Solid NMR was collected by Brucker AVANCE NEO 400. Raman spectra were recorded by Thermo Scientific DXR Raman Microscope. Thermogravimetric analysis (TGA) was performed by Netzsch STA449F3. Scanning electron micrograph was taken by JEOL JSM-7900F. In situ

ultraviolet-visible (UV-Vis) spectra were recorded using a homemade cell with ~3 mL electrolyte by Agilent G6860A. The electronic conductivity of Li_2Q was tested by digital multimeter. which is relatively high. X-Ray diffraction (XRD) patterns were collected by Rigaku MimFLex600. The Li_2Q electrode for in situ XRD test consists of Li_2Q and multi-walled carbon nanotubes (MWCNTs) with a mass ratio of 8:2, and pristine separator was used to avoid the interference of diffraction peaks of ZIF-7.

Electrochemical measurements

Li_2Q powder and MWCNTs were weighed with the mass ratio of 5:3 and then ball-milled for 4 hours at a rotate speed of 500 rpm. The ball-milled mixture, Ketjen black, and PVDF binder were weighed with the mass ratio of 8:1:1, that is, the mass ratio of Li_2Q, conductive carbon, and PVDF is 5:4:1. The aforementioned mixture was dispersed by using NMP as the solvent. The resulting slurry was cast on Al foil, followed by heating at 100 °C for 24 hours and cut into a circular disc with a diameter of 10 mm. CR-2032 coin cell was assembled by using Li_2Q as the cathode, lithium disk as the anode, pristine Celgard or ZIF-modified Celgard as the separator, 20 μL 1 mol kg^{-1} LiTFSI in EC/DMC (1:1 vol%) as the electrolyte. CV tests were performed via CHI 660E electrochemical workstation (ChenHua, Shanghai). The galvanostatic charge/discharge tests at different current rates (1 C = 439 mA g^{-1}) of batteries in the voltage range of 1.5−3.5 V (vs. Li^+/Li) were conducted by using Land CT2001A.

Density Functional Theory (DFT) Calculation Details

Density functional theory (DFT) calculations were performed within the projector augmented wave (PAW) method as implemented in Vienna Ab-initio Simulation Package (VASP).[2–4] The Perdew-Burke-Ernzerhof (PBE) functional based on the generalized gradient approximation (GGA) was adopted to describe the electron exchange and correlation effects.[5] The cutoff energy was set to 450 eV. The convergence criteria for the energy and force were set to 10^{-5} eV and 0.02 eV $Å^{-1}$, respectively. The long-range dispersion correction for the van der Waals interaction was implemented through the DFT-D3 method in all calculations.[6] The bulk Li_2Q was modeled with a 1 × 1 × 2 supercell while the bulk BQ was modeled with a 2 × 2 × 2 supercell. The diffusion energy barriers were obtained based on the climbing-image nudged elastic band (CI-NEB) method.

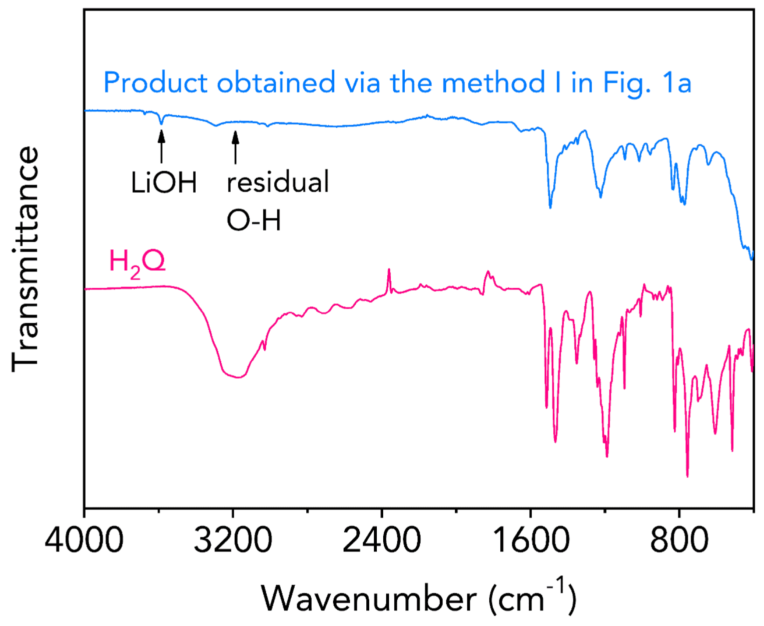

Figure S1. IR spectra of the raw material (H_2Q) and the product obtained via the first method (method I) in Fig. 1a. There are peaks assigned to LiOH and residual O−H in the product. Note that the IR spectrum of H_2Q was copied from the result in Fig. 2a.

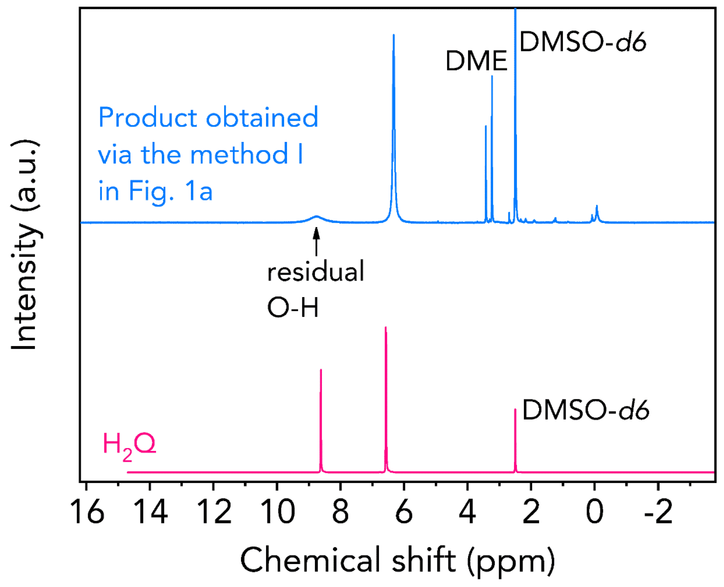

Figure S2. ^{1}H NMR spectra of the raw material (H_2Q) and the product obtained via the first method (method I) in Fig. 1a. The broad peak at 8.7 ppm can be attributed to the residual O−H. The integral area ratio of 8.7 ppm to 6.3 ppm is 0.21: 1.

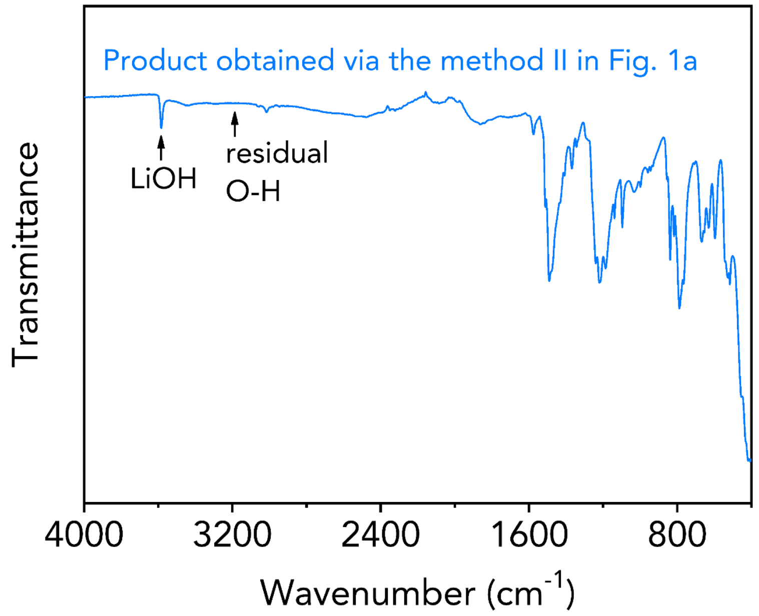

Figure S3. IR spectrum of the product obtained via the second method (method II) in Fig. 1a. There are peaks assigned to LiOH and residual O–H in the obtained product.

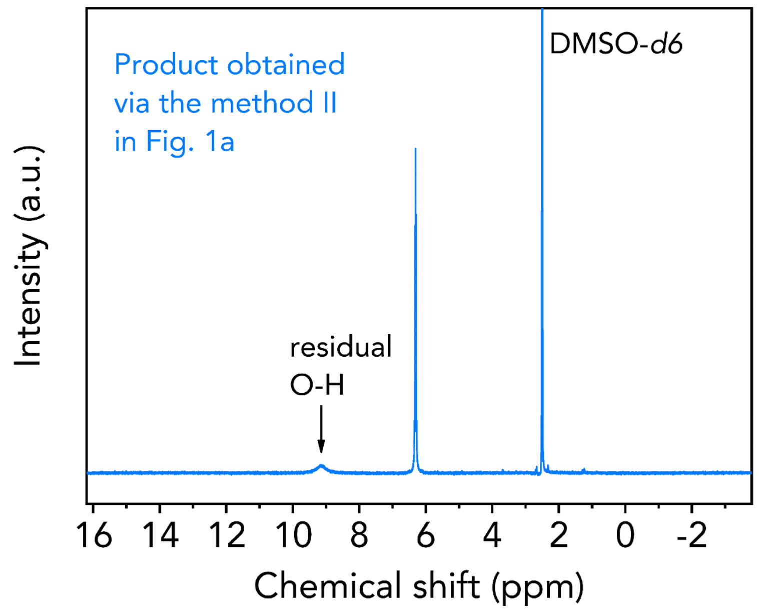

Figure S4. ^1H NMR spectra of the product obtained via the second method (method II) in Fig. 1a. The broad peak at 9.1 ppm can be attributed to the residual O–H. The integral area ratio of 9.1 ppm to 6.3 ppm is 0.25: 1.

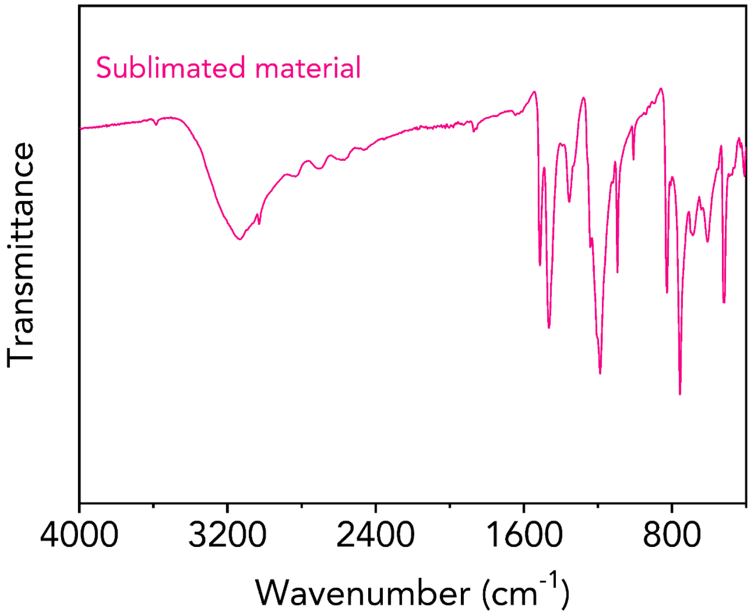

Figure S5. IR spectrum of the sublimated material. This spectrum is similar to that of H_2Q raw material.

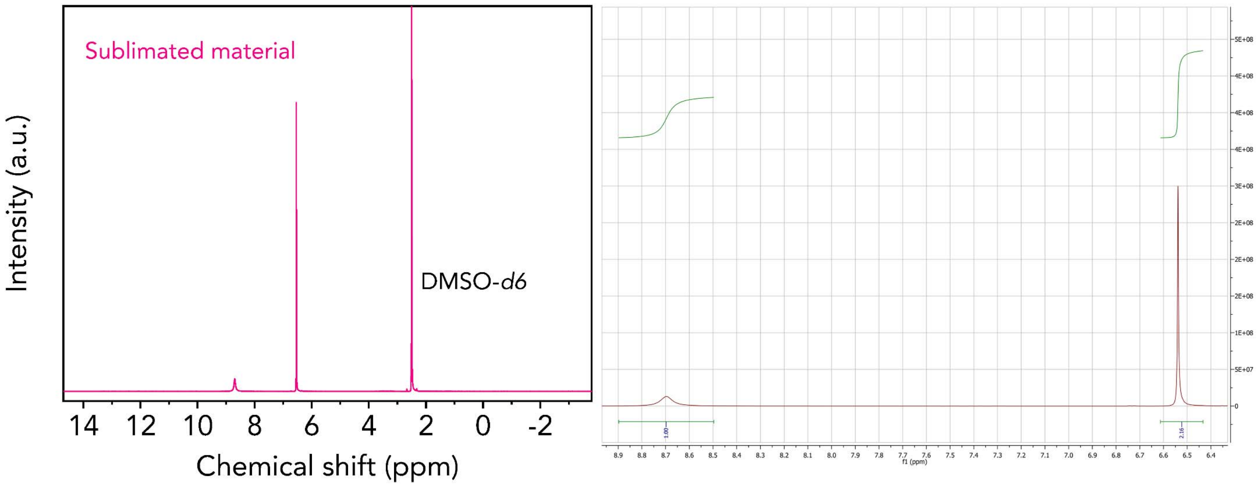

Figure S6. ^1H NMR spectrum of the sublimated material. Note that the inset on the right shows that the integral area ratio of 8.70 ppm to 6.54 ppm is 1: 2.16. Thus, the IR and ^1H NMR spectra verify that the sublimated material is indeed H_2Q.

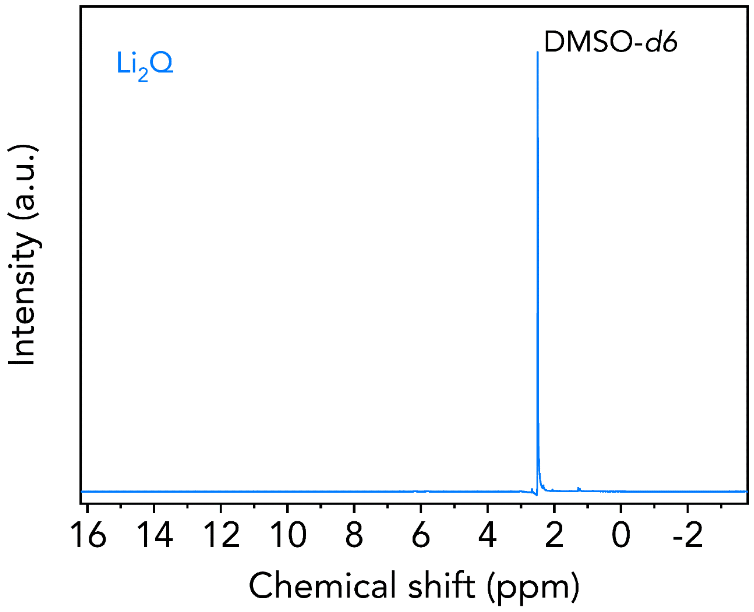

Figure S7. Liquid-state ^{1}H NMR spectrum of Li_2Q product. Except for the DMSO-*d6* solvent, no perk was observed, indicating that the prepared Li_2Q shows limited solubility in high-polarity DMSO-*d6*.

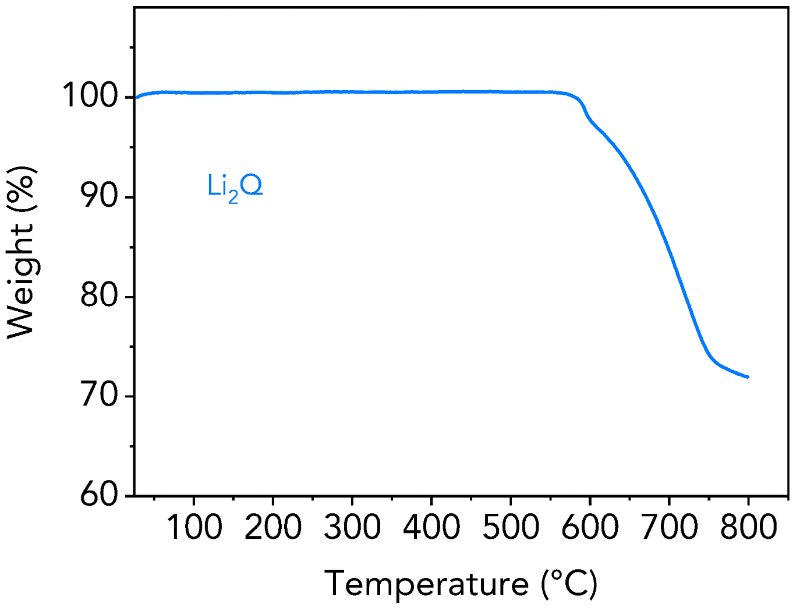

Figure S8. TG curve of the prepared Li_2Q in the temperature range of 25−800 °C at a heat rate of 5 °C min^{-1} (Ar atmosphere). The weight loss of Li_2Q starts at ~568 °C.

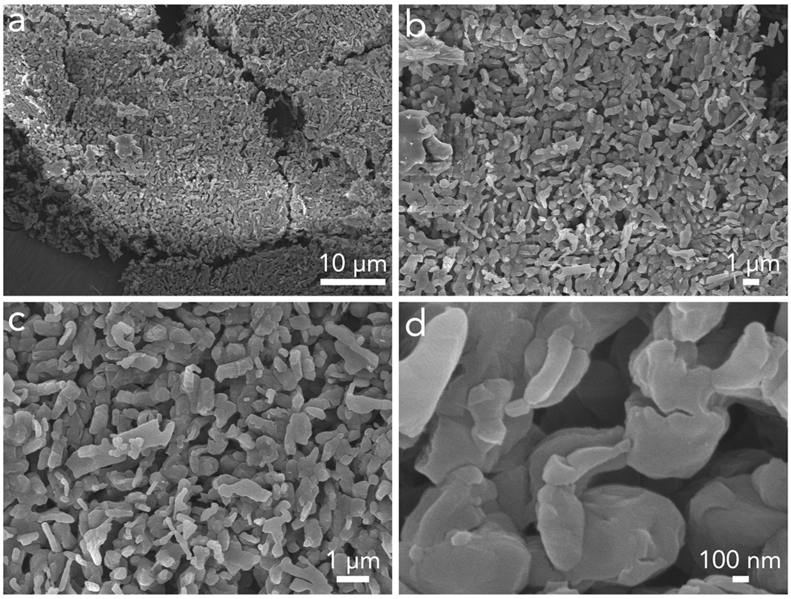

Figure S9. SEM images with low and high resolution of the prepared Li_2Q.

Table S1. Crystal parameters of Li_2Q.

Crystal system: *orthorhombic*						
a	b	c	V	α	β	γ
11.16860 Å	10.40017 Å	4.71943 Å	548.1870 Å^3	90°	90°	90°

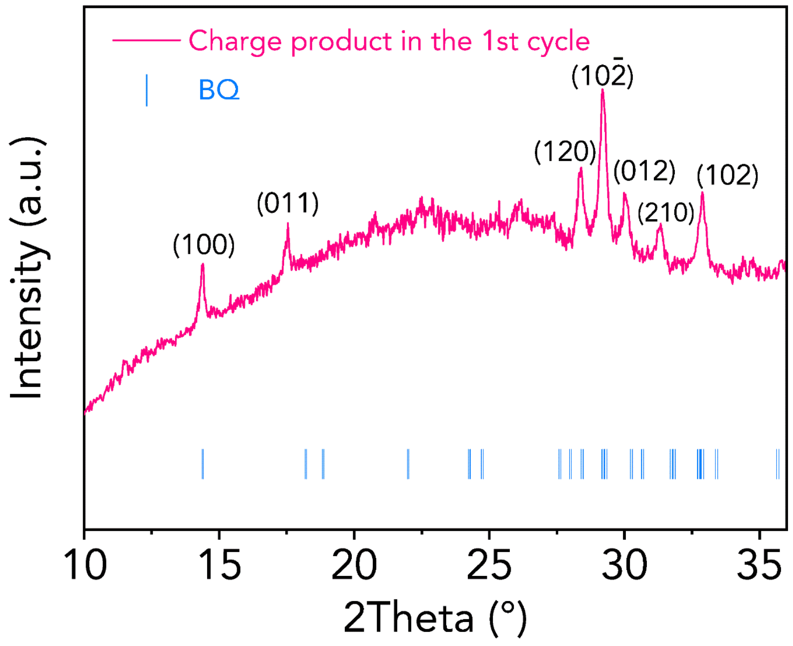

Figure S10. XRD pattern of the charge product in the 1st cycle. The Bragg positions of BQ were obtained from Rietveld refinement and the corresponding crystal structure of BQ is shown in Fig. S11.

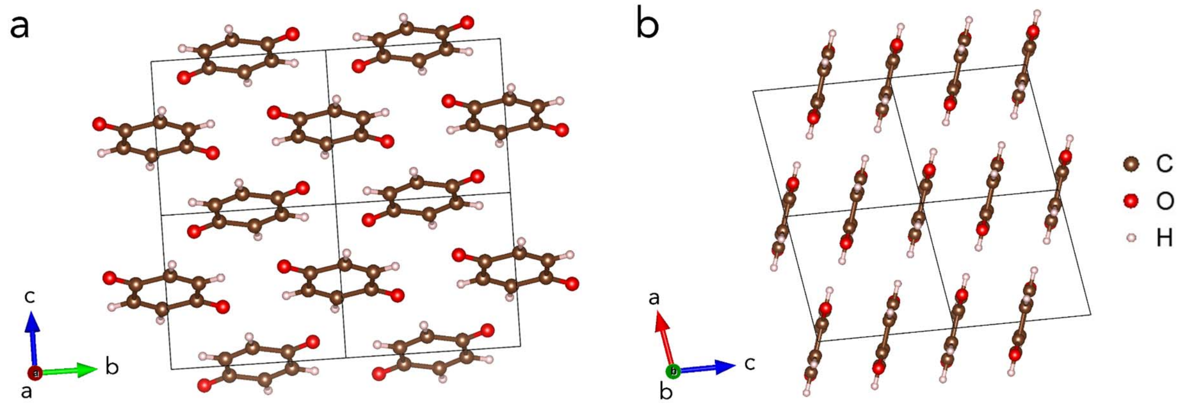

Figure S11. Crystal structure of the charge product (BQ) obtained from Rietveld refinement.

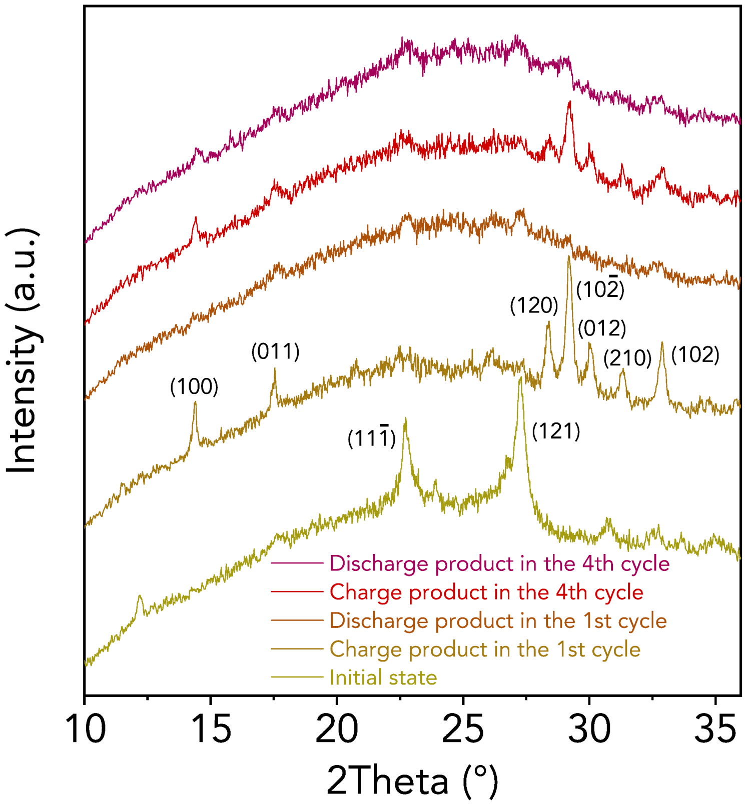

Figure S12. In situ XRD patterns of five selected states, including initial state, fully charged and discharged states in the 1st cycle, and fully charged and discharged states in the 4th cycle.

Note that the XRD patterns of initial state, fully charged and discharged states in the 1st cycle were copied from the results in Fig. 3c, and the XRD pattern of the fully charged state in the 1st cycle was also shown in Fig. S10.

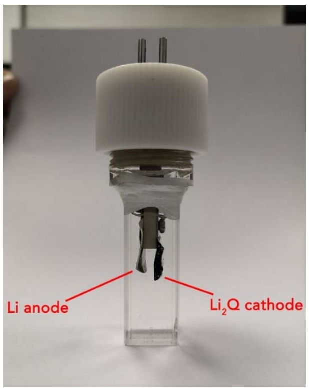

Figure S13. Optical photograph of the homemade cell for in situ UV-vis spectra tests. The separator will be used to fully wrap the Li_2Q cathode during tests.

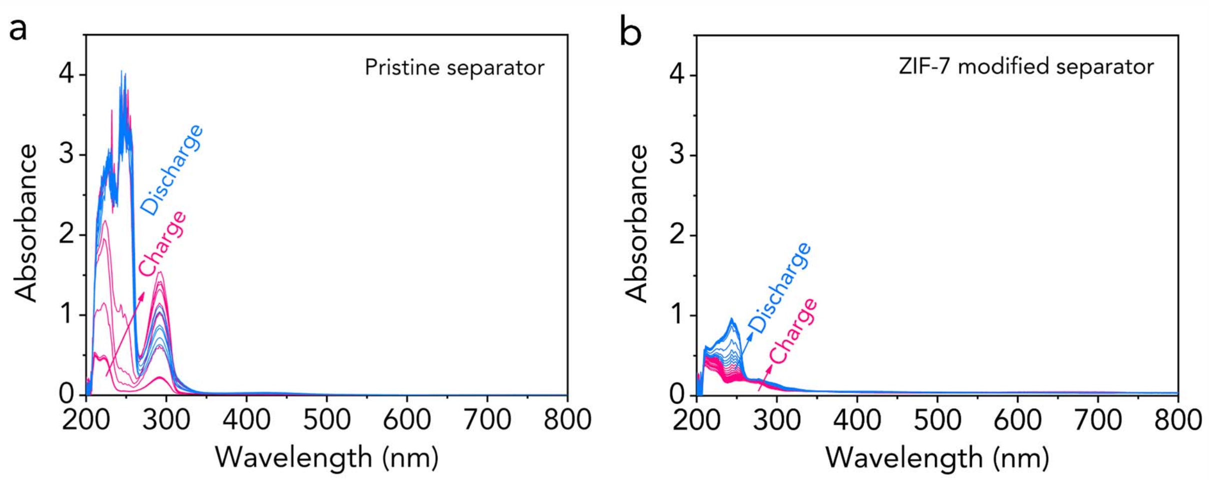

Figure S14. Detailed curves of the in situ UV-vis spectra of Li_2Q batteries with (a) a pristine separator and (b) a ZIF-7 modified separator.

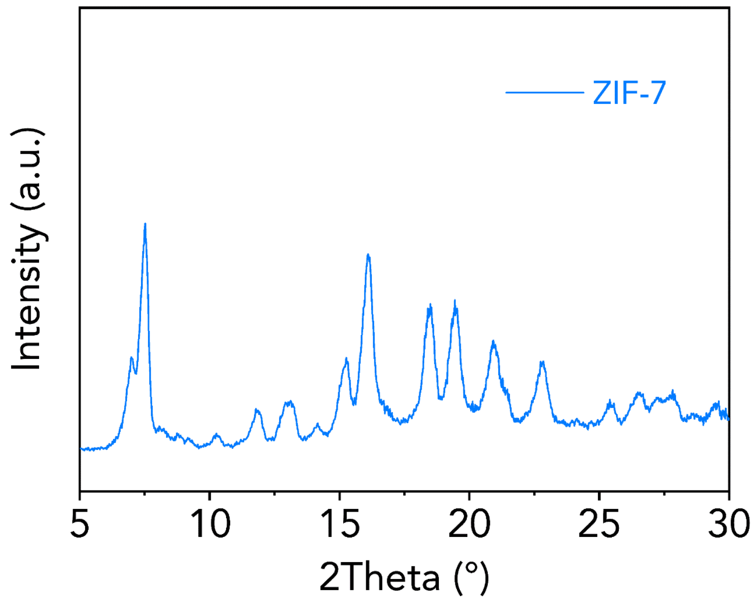

Figure S15. XRD pattern of the prepared ZIF-7.

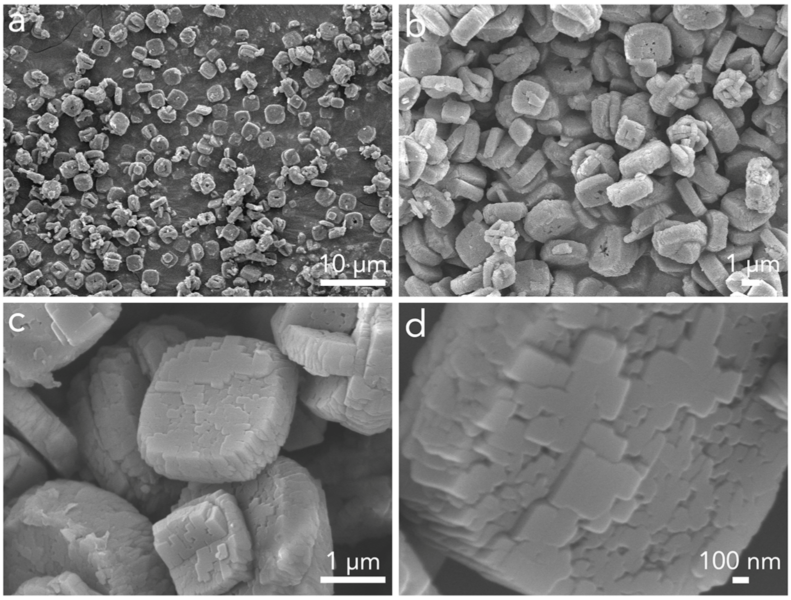

Figure S16. SEM images with low and high resolution of the prepared ZIF-7.

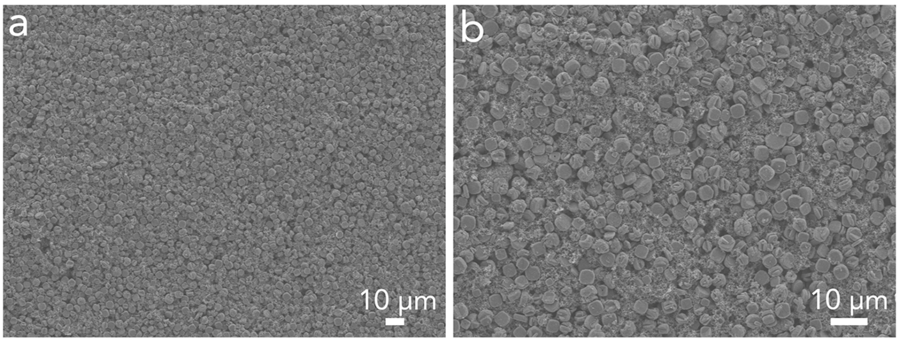

Figure S17. SEM images of the ZIF-7 modified Celgard separator.

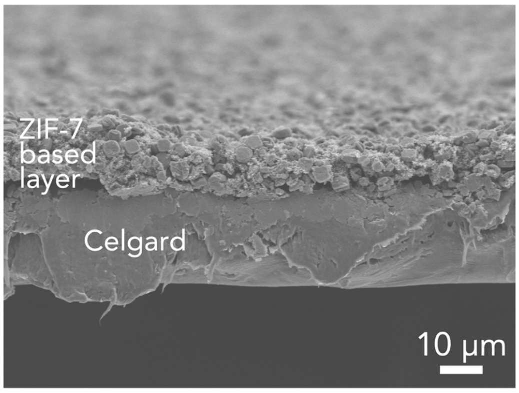

Figure S18. Cross-sectional SEM image of the ZIF-7 modified Celgard separator. The thickness of the ZIF-7 based layer is ~10 μm.

References

[1] Z. Chang, Y. Qiao, H. Deng, H. Yang, P. He, H. Zhou. *Joule* **2020**, *4*, 1776−1789.

[2] G. Kresse, J. Furthmüller. *Phys. Rev. B: Condens. Matter Mater. Phys.* **1996**, *54*, 11169−11186.

[3] G. Kresse, J. Furthmüller. *Comput. Mater. Sci.* **1996**, *6*, 15−50.

[4] G. Kresse, D. Joubert. *Phys. Rev. B: Condens. Matter Mater. Phys.* **1999**, *59*, 1758−1775.

[5] J. P. Perdew, K. Burke, M. Ernzerhof. *Phys. Rev. Lett.* **1996**, *77*, 3865−3868.

[6] S. Grimme, J. Antony, S. Ehrlich, H. Krieg. *J. Chem. Phys.* **2010**, *132*, 154104.
